# Sustainable iodine contrast media for a healthy future (SUSTAINED): a global survey of practice

**DOI:** 10.1186/s13244-026-02392-4

**Published:** 2026-10-11

**Authors:** Andrew England, Yasser H. Hadi, Helle Precht, Dávid Sipos, Nejc Mekiš, Patrizia Cornacchione, Anastasia Sarchosoglou, Tiina Nousiainen, Altino Cunha, Caroline Justich, Cheryl Cruwys, Dominique Carrié, Mark F. McEntee

**Affiliations:** 1 Discipline of Medical Imaging and Radiation Therapy, School of Medicine and Health, University College Cork, Cork, Ireland; 2 Health Sciences Research Centre, UCL University College, Odense, Denmark; 3 Department of Medical Imaging and Intervention, King Abdullah Medical City (KAMC), Makkah, Saudi Arabia; 4 Department of Regional Health Research, University of South Denmark, Odense, Denmark; 5 Department of Radiology, Lillebaelt Hospital, University Hospitals of Southern Denmark, Kolding, Denmark; 6 Department of Medical Imaging, Faculty of Health Sciences, University of Pécs, Pécs, Hungary; 7 Medical Imaging and Radiotherapy Department, Faculty of Health Sciences, University of Ljubljana, Ljubljana, Slovenia; 8 Fondazione Policlinico Universitario A. Gemelli IRCCS, Rome, Italy; 9 Università Cattolica del Sacro Cuore, Rome, Italy; 10 General Oncological Hospital of Kifissia, Kifissia, Greece; 11 Department of Diagnostic Services, Hospital Nova, Wellbeing Services County of Central Finland, Jyväskylä, Finland; 12 European Federation of Radiographer Societies (EFRS), Porto, Portugal; 13 Be Accepted GmbH, Vienna, Austria; 14 DenseBreast-Info Inc, Deer Park, NY USA; 15 Centre de Radiologie et d’Imagerie Medicale du parc, Toulouse, France; 16 Faculty of Health Sciences, University of Sydney, Sydney, Australia

**Keywords:** Contrast agent, Safety (patient), Management (waste), Environmental impact

## Abstract

**Objectives:**

To investigate healthcare professionals’ knowledge, attitudes, and practices (KAPs) of the environmental impact and sustainability of iodinated contrast media (ICM).

**Materials and methods:**

A cross-sectional web-based survey was distributed to members of professional societies and advertised via social media between June and December 2024. The questionnaire covered themes including sustainability attitudes, reduction strategies, waste management, patient awareness, and institutional policies. Data were analysed using descriptive statistics, chi-square tests, and analysis of variance (ANOVA).

**Results:**

231 respondents from 44 countries completed the survey, primarily diagnostic radiographers (59%) and radiologists (38%). Most respondents (60%) reported that their institutions had sustainable ICM policies, and there was a significant association between institutional size and alignment with sustainability goals $\left({X}^{2}=14.2,{p}=0.003\right)$. Adoption of reduction strategies such as protocol optimisation, saline chasers, and multi-patient systems varied significantly across institutions $\left({X}^{2}=18.5,{p}=0.001\right)$, with younger professionals (25–44 years) more engaged $\left({ANOVA},{F}=5.62,{p}=0.001\right)$. Respondents reported waste management practices being inconsistently applied, with 26% of respondents reporting “never engaging” $\left({X}^{2}=19.6,{p} \, < \,0.001\right)$. Patient awareness was perceived as low, 47% “strongly disagreeing” that patients understood the environmental impact of ICM use $\left({X}^{2}=16.9,{p}=0.002\right)$. Variability in KAPs across European countries was minimal $\left({s}^{2}=0.0068\right)$.

**Conclusions:**

This survey highlights widespread professional awareness of ICM sustainability issues but reveals variability in institutional practices, limited patient awareness, and inconsistent waste management. Findings emphasise the importance of stronger institutional policies, targeted education, and international collaboration to promote sustainable ICM use.

**Key Points:**

***Question*** How do healthcare professionals perceive and implement sustainable practices in the use of iodinated contrast media (ICM) within clinical practice?

***Findings*** While many institutions endorse sustainability, notable gaps exist in waste management, patient and practitioner education, and consistency of reduction strategies.

***Critical relevance statement*** Strengthening institutional frameworks and professional and patient engagement can enhance sustainable ICM use, safeguard resources, and mitigate environmental impact.

**Graphical Abstract:**

**Figure:**
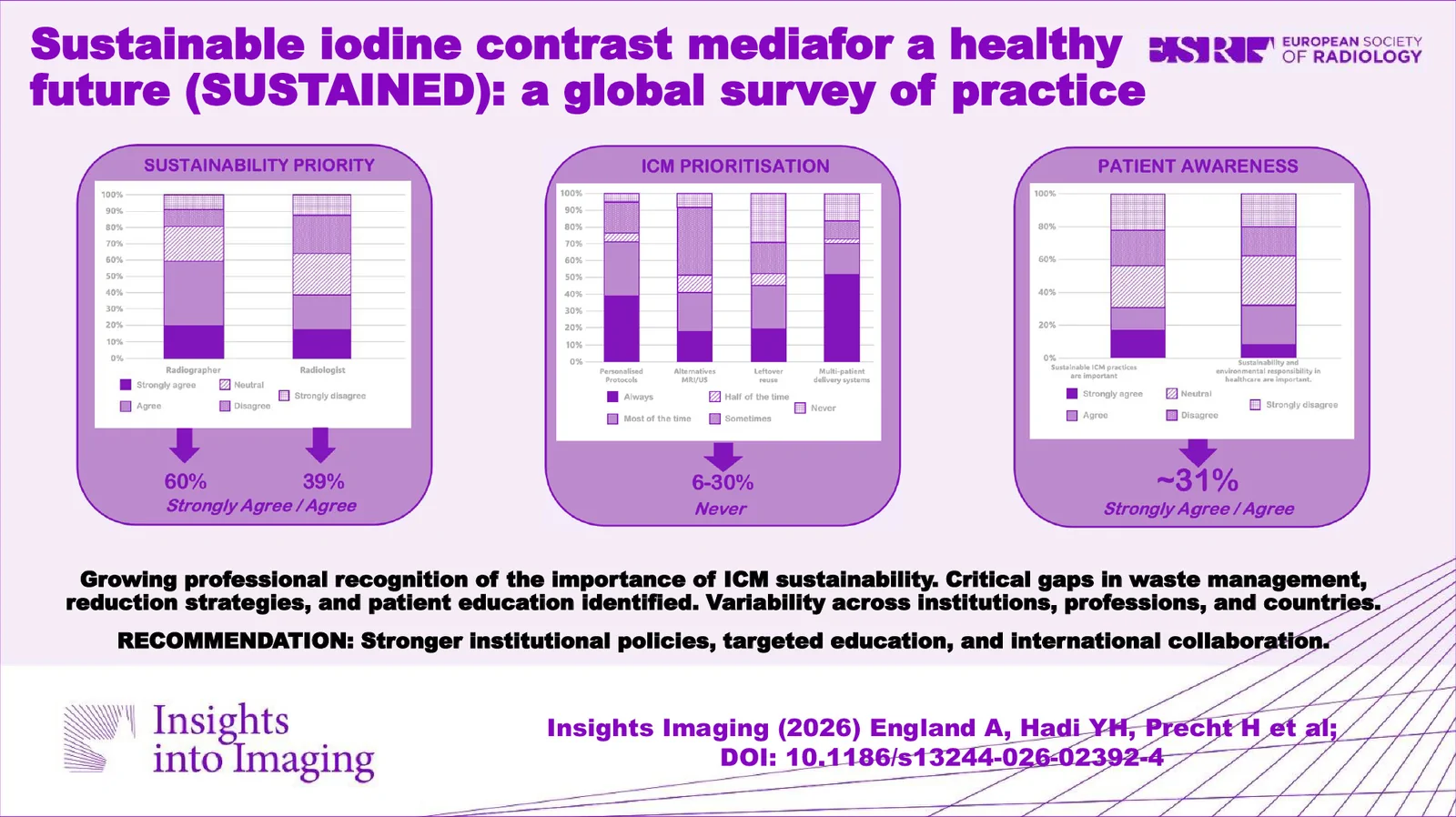

## Introduction

Iodinated contrast media (ICM) are the most commonly administered pharmaceuticals in medical imaging and significantly improve the diagnosis and evaluation of a wide range of pathophysiological conditions [1]. Due to the rapid growth of medical imaging over the past few decades [2], particularly in CT [3], there has been a substantial increase in the demand for ICMs [4]. For example, studies have suggested that the use of ICMs in CT has more than doubled and is currently used in approximately 50% of all CT examinations [5, 6]. Dekker and colleagues estimate that over 10 million litres of ICMs are required each year globally [7]. As with other aspects of healthcare, the rising demand for medical imaging is well noted. ICM requirements are growing, and the literature reports supply issues [8]. Global iodine reserves of 6.3 million tonnes have been reported, with current consumption rates in the region of 0.5% per annum [9]. According to GE HealthCare, current trends indicate that the clinical demand for ICMs will eventually outstrip supply [10], with some models predicting that this could be within the next 170 years unless iodine is extracted from seawater, which is currently challenging [11].

Supply issues when combined with the continuing rise in iodine utilisation has made ICM sustainability a frequently debated topic, for example, at the 2025 European Congress of Radiology with a theme of ‘Planet Radiology’. Supplies are not the only issue; a further concern is the environmental impact of waste ICMs or related products. Wastage from single-use items [12], leftover ICMs [13], with discharge recorded in waste and drinking water [14] are further key environmental themes, as is the carbon cost of ICM manufacture [15].

Sustainability within radiology is becoming increasingly more important among professional societies. Recently this was evident in the joint position paper and call to action article by the American College of Radiology, Asian Oceanian Society of Radiology, African Society of Radiology, Canadian Association of Radiologists, Colegio Interamericano de Radiología, European Society of Radiology, Egyptian Society of Radiology and Nuclear Medicine, International Society for Strategic Studies in Radiology, International Society of Radiology, Royal Australian and New Zealand College of Radiologists and Radiological Society of North America [16]. Global work continues to make radiology more sustainable; in 2024, at the Radiology R7 Summit, a framework for environmentally sustainable radiology was developed, which included a focus on reducing the use of finite resources and waste [17]. A further option is the Climate Resilient and Environmentally Sustainable Radiology Framework, which is based on five pillars of climate resilience capacity—threshold, coping, recovery, adaptive, and transformative [18].

A growing number of initiatives seeking to tackle the issue of ICM sustainability have been reported [1, 19]. Despite this, many questions remain unanswered, and uncertainties exist. The aim of this study is to develop an understanding of the knowledge, attitudes and practices of those involved in medical iodinated contrast media administration. Such information would be advantageous in identifying research priorities and developing strategies to help ensure sustainable ICM supplies and environmentally responsible disposal.

## Materials and methods

### Questionnaire formulation

The study was based on a survey of iodinated contrast media sustainability practises among relevant stakeholders, those involved in any aspect of ICM administration. Ethical approval to distribute the survey was obtained from the Social Research Ethics Committee of University College Cork (REF 2024-086). A draft questionnaire was formulated by the lead author (A.E.) and was broadly based on the previous work of Rengier et al [12]. Survey themes included attitudes towards ICM sustainability, strategies to reduce ICM consumption, ICM and consumable waste management practices, perceived patient awareness and ICM sustainability priorities. A peer group of three medical imaging professionals provided content validation. The online version of the questionnaire was implemented within Qualtrics (Qualtrics Inc., Victoria, AU). A pilot test was performed within the research team and resulted in only minor typographical changes. Allowing for the use of branching questions, the survey required up to 34 responses and took approximately 5 min to complete (Supplementary File S1). Duplicate and incomplete responses were removed during data cleaning.

### Questionnaire distribution

The target audience for dissemination was multidisciplinary professionals involved in the administration of iodinated contrast media. Professions could include, but were not limited to, radiologists, radiographers, cardiologists and vascular surgeons. A series of social media posts (LinkedIn, Facebook, Twitter [X] and Instagram) were created and disseminated via professional networks of the research team, the European Society of Radiology (ESR) and the European Federation of Radiographer Societies (EFRS). An electronic link to the questionnaire was further distributed by email to the membership of the EFRS and ESR. Both the EFRS and ESR would have access to a multidisciplinary membership, but the ESR would predominantly focus on radiologists and the EFRS radiographers. Data available in 2024 indicated that the membership of the ESR was approximately 142,000 and that the EFRS represented over 100,000 radiographers. Snowballing techniques were further used to invite potential participants to share the survey link with colleagues. In total, the survey was open for 6 months (June to December 2024), and frequent reminders were circulated using social media posts at approximately fortnightly intervals.

### Analysis of responses

Responses were collated within the online survey site and with Microsoft Excel (Microsoft Corp, Redmond, WA). Duplicated responses and redundant blank forms were screened and removed. Quantitative data were assessed visually for normality and also using the Shapiro–Wilk test. Descriptive statistics, including frequencies and percentages, were used to summarise the demographic characteristics and response patterns. Chi-square tests were employed to examine associations between institutional size, professional roles, and countries with sustainability-related practices and perceptions, with a significance level set at *p* < 0.05. Analysis of variance (ANOVA) was used to compare age-group differences, followed by Tukey post hoc tests to identify specific group differences. Prior to conducting statistical testing, appropriate assumptions of the data were evaluated in line with the requirements of the individual tests. Variability in KAPs across European countries was assessed by generating a composite KAP score for each respondent, which was created using numerically coded Likert-scale items on knowledge, attitudes, and practices. These scores were then aggregated at the country level to obtain mean KAP scores for each European country. The sample variance of these country-level mean KAP scores was reported. Statistical analyses were performed in SPSS V23 (IBM Corp., Armonk, NY) and graphical figures were created using Microsoft Excel (Microsoft Corp, Redmond, WA).

## Results

### Demographics

A total of 231 respondents participated in the study, providing broad representation across professional backgrounds and institutional affiliations (Supplementary File S2). Most were diagnostic radiographers (136, 58.9%), followed by radiologists (87, 37.7%). Cardiologists, therapeutic radiographers, and industry representatives together comprised 3.4% (*n* = 8) of the sample. Almost all 224 (97%) respondents indicated that they were directly involved in ICM administration. Respondents represented 44 countries, with the United Kingdom and Hungary contributing the largest shares (each 17.3%), followed by Ireland (8.2%) (Table 1). Other countries contributed varying numbers, highlighting the study’s extensive geographical reach.

**Table 1:** Frequency of survey responses per country with ≥ 4 respondents

Country	Number of respondents (*n*)	Percentage of respondents (%)
United Kingdom of Great Britain and Northern Ireland	40	17.3
Hungary	40	17.3
Other	38	16.4
Ireland	19	8.2
Italy	14	6
Spain	12	5.1
Germany	11	4.7
Greece	9	3.8
Portugal	8	3.4
Denmark	7	3
Netherlands	6	2.5
Switzerland	6	2.5
Belgium	5	2.1
Australia	4	1.7
Ghana	4	1.7
Romania	4	1.7
Finland	4	1.7
Total	231 respondents	100
	44 countries	

### Demographic information of respondents

The respondents’ demographic profile highlighted a balanced gender distribution, with 118 (51.1%) identifying as female and 109 (47.2%) as male, while four (1.7%) chose not to disclose their gender. Age groups were predominantly 35–44 years (72, 31.2%) and 25–34 years (59, 25.5%), with fewer respondents under 25 years (9, 3.9%) or over 55 years (34, 14.7%). Detailed age-group distribution and their opinions on minimising iodine dosage are shown in Table 2. Among the institutions, large public hospitals employed the majority (88, 38.1%), followed closely by medium public hospitals (76, 32.9%). Additionally, smaller public and private institutions made up the remainder, reflecting participation from diverse institutional sizes (Table 3). These findings underline the engagement of professionals across a broad spectrum of demographics and institutional frameworks.

**Table 2:** Age distribution and opinions on minimising iodine dose in imaging procedures

	Giving the lowest possible iodine dose to a patient is a priority.						
	Strongly agree	Agree	Neither agree/disagree	Disagree	Strongly disagree	Not applicable/don’t know	Total (%)
**Age (years)**							
Under 18	0 (0)	1 (0.4)	0 (0)	0 (0)	0 (0)	0 (0)	1 (0.4)
18–24	5 (2.2)	3 (1.3)	0 (0)	0 (0)	0 (0)	0 (0)	8 (3.5)
25–34	14 (6.1)	31 (13.4)	10 (4.3)	3 (1.3)	1 (0.4)	0 (0)	59 (25.5)
35–44	22 (9.5)	30 (13)	13 (5.6)	4 (1.7)	3 (1.3)	0 (0)	72 (31.2)
45–54	19 (8.2)	24 (10.4)	5 (2.2)	6 (2.6)	3 (1.3)	0 (0)	57 (24.7)
55–64	13 (5.6)	7 (3.0)	3 (1.3)	2 (8.7)	2 (0.9)	0 (0)	27 (11.7)
65+	2 (0.9)	3 (1.3)	0 (0)	1 (0.4)	0 (0)	1 (0.4)	7 (3)
Total (%)	75 (32.5)	99 (42.9)	31 (13.4)	16 (6.9)	9 (3.9)	1 (0.4)	231 (100)

**Table 3:** Perceptions of iodinated contrast media sustainability by institution and profession

	Iodinated contrast media sustainability is a priority within my workplace.						
	Strongly agree	Agree	Neither agree/disagree	Disagree	Strongly disagree	Not applicable/don’t know	Total (%)
**Workplace/institution categories**							
Large-sized public hospital (> 500 beds)	19	16	26	16	7	4	88 (38.1)
Medium-sized public hospital (100–500 beds)	12	28	20	10	6	0	76 (32.9)
Small-sized public hospital (< 100 beds)	3	11	2	0	0	0	16 (6.9)
Large-sized private hospital (> 500 beds)	1	2	0	0	1	0	4 (1.7)
Medium-sized private hospital (100–500 beds)	3	1	2	1	0	0	7 (3)
Small-sized private hospital (< 100 beds)	0	1	0	0	0	0	1 (0.4)
Combined public/private hospital	0	0	1	0	1	0	2 (0.9)
Academic centre, i.e., University	0	10	3	4	4	0	21 (9.1)
Standalone imaging facility, i.e., community diagnostic hub	3	6	3	2	2	1	16 (6.9)
Total (%)	41 (17.7)	75 (32.5)	56 (24.2)	33 (14.3)	21 (9.1)	5 (2.2)	231 (100)
**Profession**							
Diagnostic radiographer/radiological technologist	26	54	31	14	10	1	136 (58.9)
Therapeutic radiographer/radiation therapist/radiotherapist	0	1	2	0	0	0	3 (1.3)
Radiologist/radiology resident	15	17	23	17	11	4	87 (37.7)
Representative from the industry	0	1	0	0	0	0	1 (0.4)
Cardiologist/cardiology resident	0	2	0	2	0	0	4 (1.7)
Total (%)	41 (17.7)	75 (32.5)	56 (24.2)	33 (14.3)	21 (9.1)	5 (2.2)	231 (100)

### Institutional attitudes

**Fig. 1:**
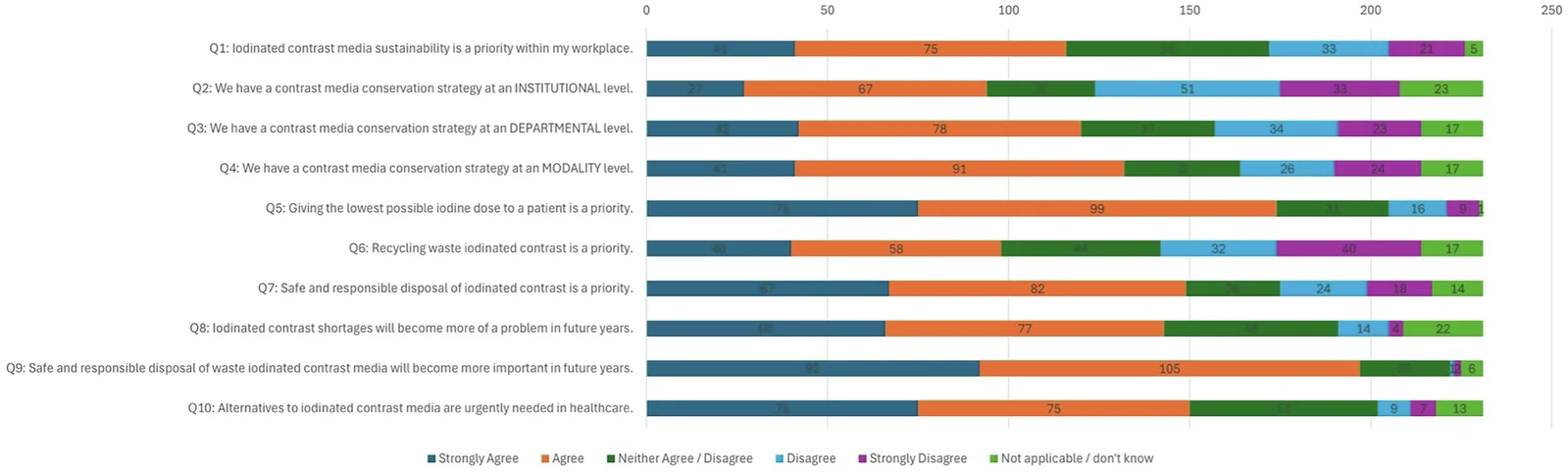
Agreement levels on workplace policies and practices related to iodinated contrast media (ICM) sustainability

Responses on institutional attitudes revealed overall positive perceptions of workplace sustainability policies (Fig. 1). A detailed breakdown by institution and profession is provided in Table 3. Approximately 50% (*n* = 116) of respondents “agreed” or “strongly agreed” that their institutions had sustainable policies, with diagnostic radiographers (68/136, 50%) and radiologists (34/82, 62%) expressing the highest levels of agreement. A notable 24.2% reported neutral attitudes, potentially indicating either uncertainty or insufficient communication regarding these policies. Statistical analysis using a chi-square test revealed a significant association between institutional size and alignment with sustainability goals $X^{2}\left(32,{n}=231\right)=14.2,{p}=.0.003$. Institutions with higher bed capacities, such as large public hospitals, reported stronger alignment with sustainability goals compared to smaller or mixed public/private institutions.

**Fig. 2:**
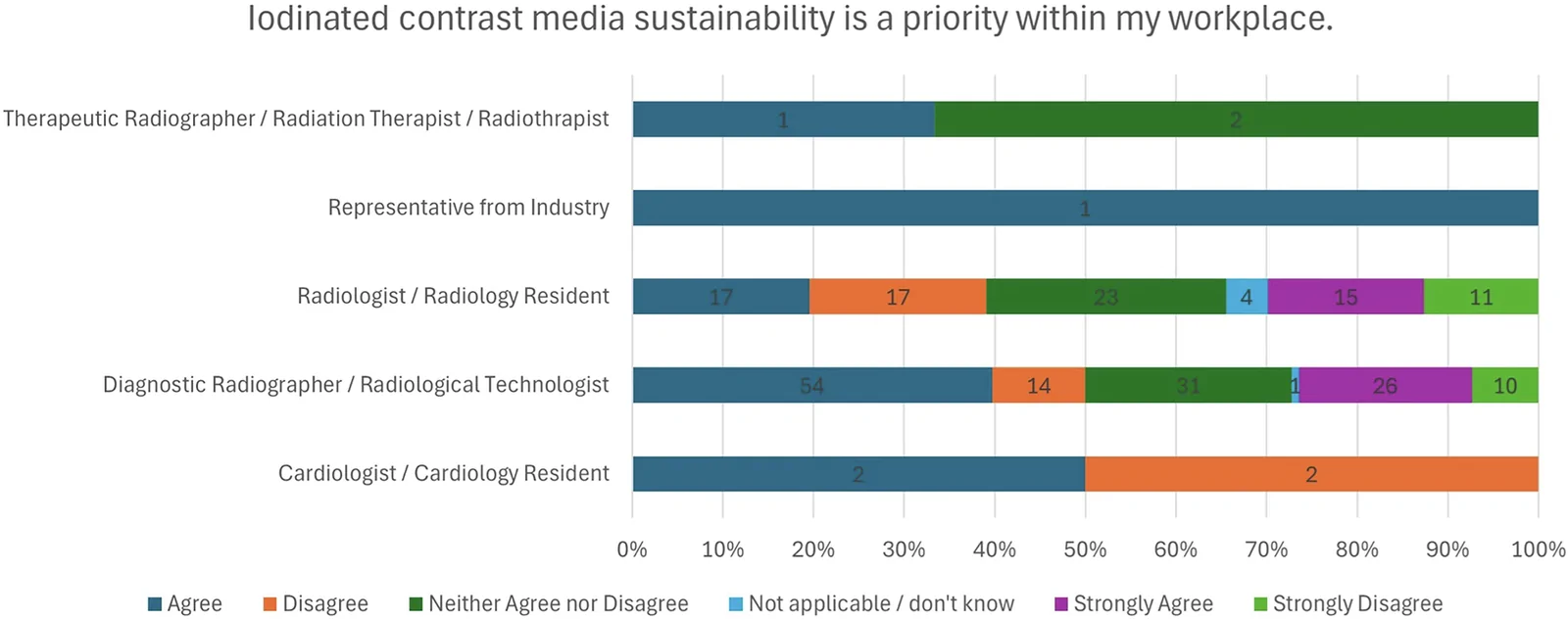
Frequency of ICM reduction strategies such as protocol optimisation, saline chasers, and multi-patient systems

Furthermore, the analysis of variability in knowledge, attitudes, and practices (KAPs) across Europe reveals a relatively low variance $\left({s}^{2}=0.0068\right)$. This finding indicates that while minor differences exist, sustainability-related practices are largely consistent across regions (Fig. 2). These results highlight that local policies, resources, and cultural attitudes may influence sustainability priorities to some extent, but they do not significantly disrupt overall alignment. Further analysis identified significant differences in KAPs across professions and countries. A chi-square test ($X^{2}\left(20,{n}=231\right)=53.51,{p}=0.02$) demonstrated notable differences across professions, reflecting varying levels of engagement and awareness among healthcare roles. Similarly, a chi-square test $\left({X}^{2}\left(15,{n}=231\right)=300.64,{p} < 0.001\right)$ confirmed substantial differences in KAPs across countries, suggesting that regional policies and healthcare infrastructure play a pivotal role in shaping practices. These findings emphasise the importance of enhancing policy visibility, fostering international collaboration, and tailoring strategies to address disparities at both professional and regional levels.

### Reduction strategies

**Fig. 3:**
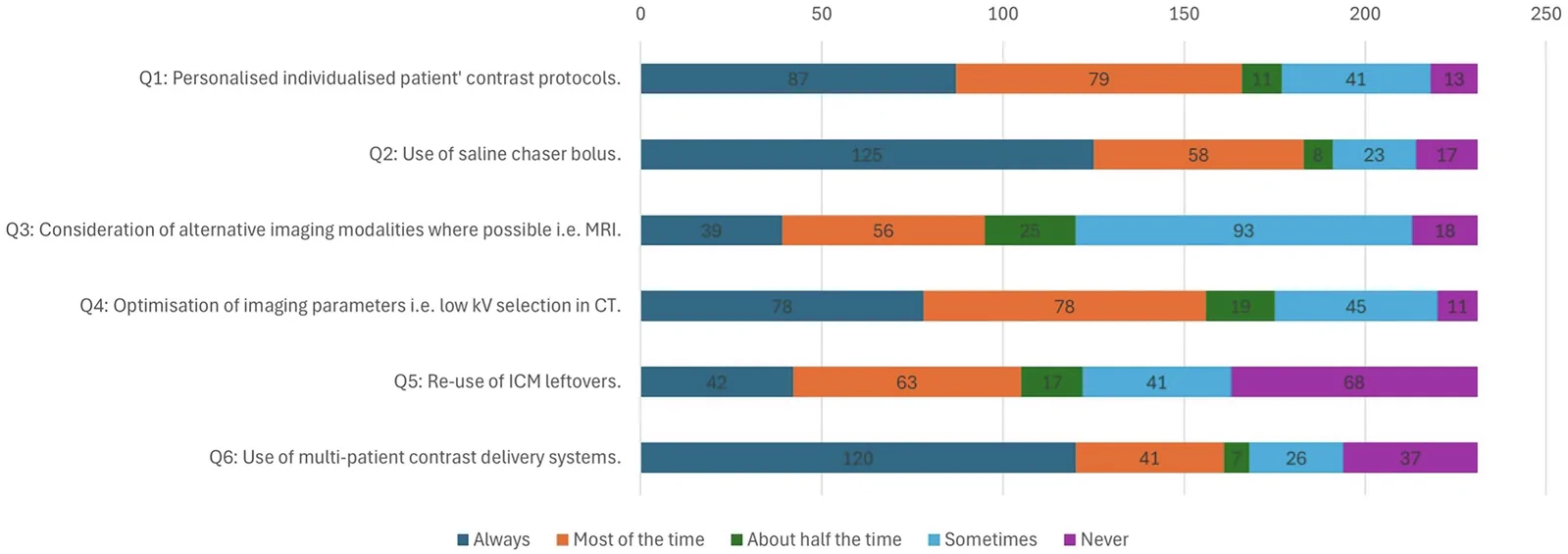
Perceptions of ICM sustainability prioritisation across professional groups

Strategies to minimise iodinated contrast media (ICM) waste demonstrated marked variation across professions and institutions (Fig. 3). Table 4 provides detailed data on strategies by institution and profession, reflecting diverse levels of commitment and resources. Large public hospitals accounted for the majority (38%) of strategies implemented, leveraging their extensive capacity and infrastructure to focus on sustainability measures. Medium public hospitals contributed significantly (33%) and often innovated within resource constraints. Diagnostic radiographers emerged as the most proactive group, actively engaging in reduction efforts and driving awareness campaigns. Conversely, smaller institutions and mixed public/private hospitals exhibited limited participation, likely hindered by resource limitations and competing priorities. Statistical analysis using a chi-square test revealed a significant association between institutional type and adoption of reduction strategies $\left({X}^{2}\left(20,{n}=231\right)=18.47,{p}=0.001\right)$. Younger professionals (25–44 years) led these initiatives, suggesting that generational attitudes toward sustainability play a pivotal role. However, participation from professionals over 45 years was noticeably lower, with an ANOVA test confirming a statistically significant difference in engagement across age groups $\left(F\left({{\mathrm{3,227}}}\right)=5.62,{p}=0.001\right)$. Post hoc Tukey analysis indicated that engagement was significantly higher among professionals aged 25–34 and 35–44 years compared with those aged 45–54 and ≥ 55 years $\left(p < 0.05\right)$, with no significant difference between the two younger groups. This confirms a greater involvement of younger professionals in reduction strategies.

**Table 4:** Perceptions of iodinated contrast media reduction strategies by institution and profession

	Personalised individualised patient contrast protocols.					
	Always	Most of the time	About half the time	Sometimes	Never	Total (%)
**Workplace/institution categories**						
Large-sized public hospital (> 500 beds)	24	33	5	18	8	88 (38.1)
Medium-sized public hospital (100–500 beds)	31	28	2	11	4	76 (32.9)
Small-sized public hospital (< 100 beds)	10	2	1	3	0	16 (6.9)
Large-sized private hospital (> 500 beds)	1	1	1	1	0	4 (1.7)
Medium-sized private hospital (100–500 beds)	3	2	0	2	0	7 (3)
Small-sized private hospital (< 100 beds)	0	1	0	0	0	1 (0.4)
Combined public/private hospital	1	1	0	0	0	2 (0.9)
Academic centre, i.e., University	11	3	2	5	0	21 (9.1)
Standalone imaging facility, i.e., community diagnostic hub	6	8	0	1	1	16 (6.9)
Total (%)	87 (37.7)	79 (34.2)	11 (4.8)	41 (17.7)	13 (5.6)	231 (100)
**Profession**						
Diagnostic radiographer/radiological technologist	57	53	6	12	8	136 (58.9)
Therapeutic radiographer/radiation therapist/radiotherapist	1	1	0	1	0	3 (1.3)
Radiologist/radiology resident	28	23	5	27	4	87 (37.7)
Representative from the industry	0	0	0	0	1	1 (0.4)
Cardiologist/cardiology resident	1	2	0	1	0	4 (1.7)
Total (%)	87 (37.7)	79 (34.2)	11 (4.8)	41 (17.7)	13 (5.6)	231 (100)

### Waste management practices

**Fig. 4:**
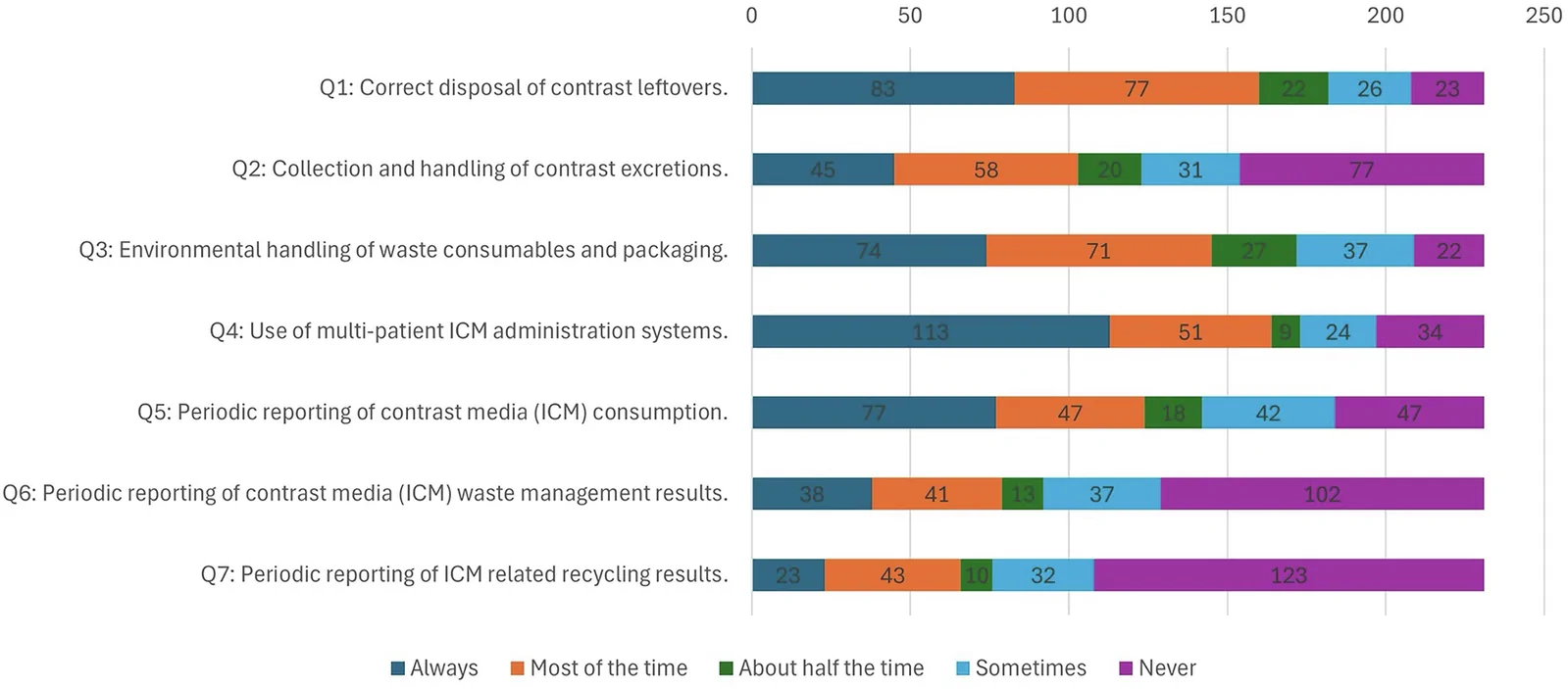
Frequency of ICM waste disposal and reporting practices

Waste management practices were evaluated across demographic and institutional categories (Fig. 4), revealing significant variability. Respondents from large public hospitals reported the highest levels of consistent engagement, with 33% indicating they always manage waste effectively. Medium public hospitals followed closely, though smaller institutions lagged considerably in their waste management practices, with a notable proportion of respondents indicating infrequent or no engagement. Alarmingly, 26% of respondents across all settings reported never engaging in waste management, highlighting a critical systemic gap. Gender analysis showed a slight dominance of female respondents (51.1%) in sustainable waste practices compared to males (47.2%), suggesting balanced but marginally higher engagement by women. Furthermore, younger professionals (25–44 years) were more likely to participate in waste management efforts than their older counterparts. Statistical analysis using a chi-square test revealed a significant association between institutional type and waste management engagement $\left(\right.{X}^{2}\,(4,\,n=231=19.62,p < 0.001)$. Additionally, an ANOVA test confirmed significant differences in waste management participation across age groups $\left(F\left(3,\,227\right)=6.78,{p} < 0.001\right)$, emphasising the influence of generational perspectives on sustainability initiatives. Post hoc Tukey analysis showed that professionals aged 25–34 and 35–44 years reported significantly higher engagement in waste management compared with those aged 45–54 and ≥ 55 years $\left(p < 0.05\right)$, with no significant difference between the younger groups.

### Perceived patient awareness

**Fig. 5:**
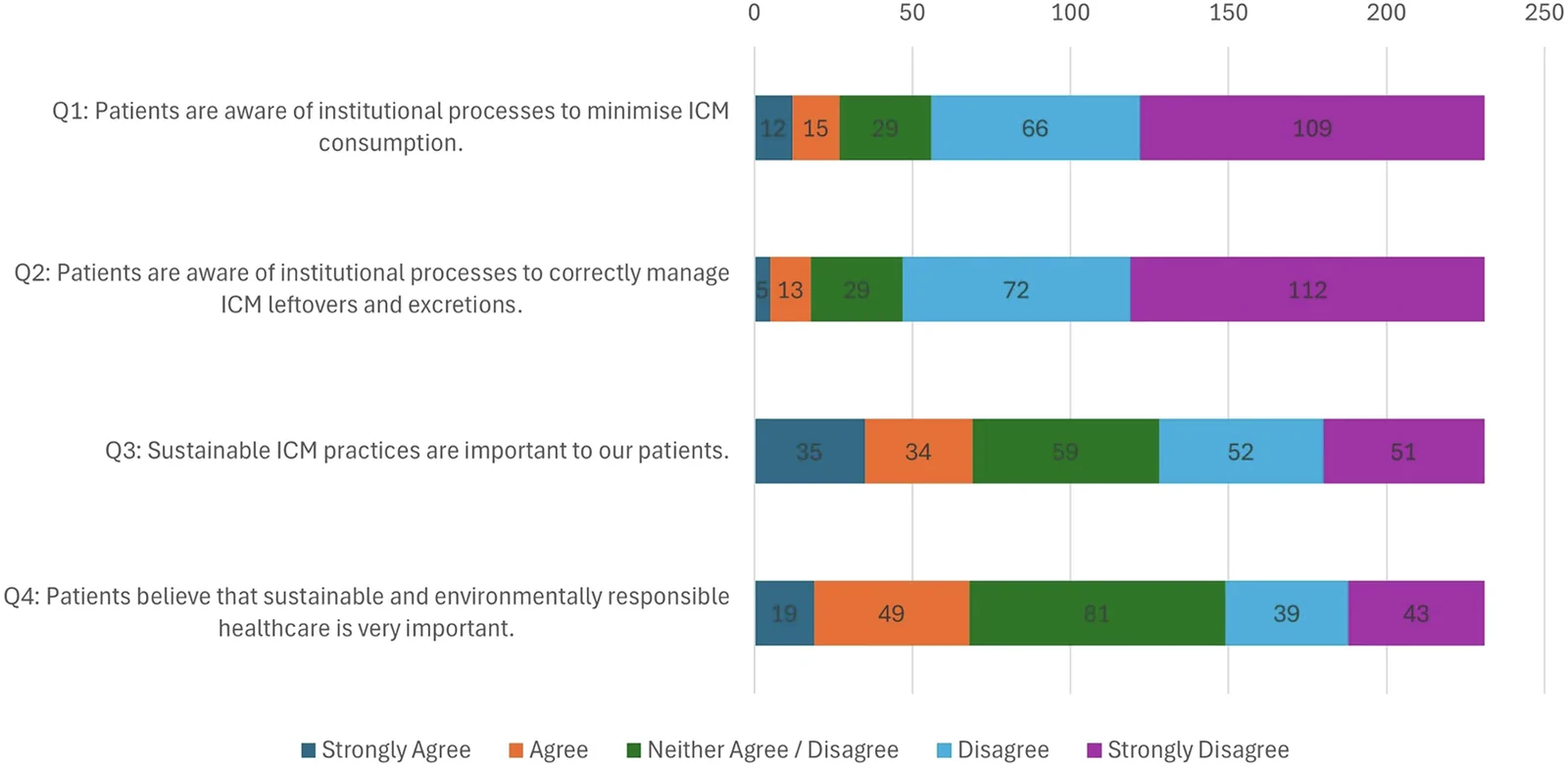
Respondents’ views on patient awareness of ICM management and sustainability

Patient awareness (healthcare professionals’ perceptions) regarding ICM sustainability was rated as low across all professional groups (Fig. 5), with marked differences in perceptions and responses. Approximately 47% of respondents strongly disagreed with the statement that patients are aware of the environmental impact of ICM use, reflecting a significant gap in patient understanding. Neutral responses accounted for 25%, possibly indicating either uncertainty or lack of direct patient engagement. Only 28% of respondents expressed agreement or strong agreement, with radiologists and diagnostic radiographers showing slightly higher levels of optimism compared to other groups. Statistical analysis using a chi-square test revealed a significant association between professional roles and perceptions of patient awareness $\left({X}^{2}\left(4,{n}=231\right)=16.94,{p}=0.002\right)$. Furthermore, an ANOVA also showed significant differences across age groups $\left(F\left({{\mathrm{3,227}}}\right)=4.14,{p}=0.007\right)$. Post hoc Tukey analysis indicated that respondents aged 25–34 and 35–44 years perceived patient awareness as lower than that of respondents aged ≥ 55 years $\left(p < 0.05\right)$, with no significant difference between the two younger groups. These findings suggest that generational attitudes may influence how professionals evaluate patients’ understanding, reinforcing the need for targeted patient education initiatives.

### Drivers of sustainability

**Fig. 6:**
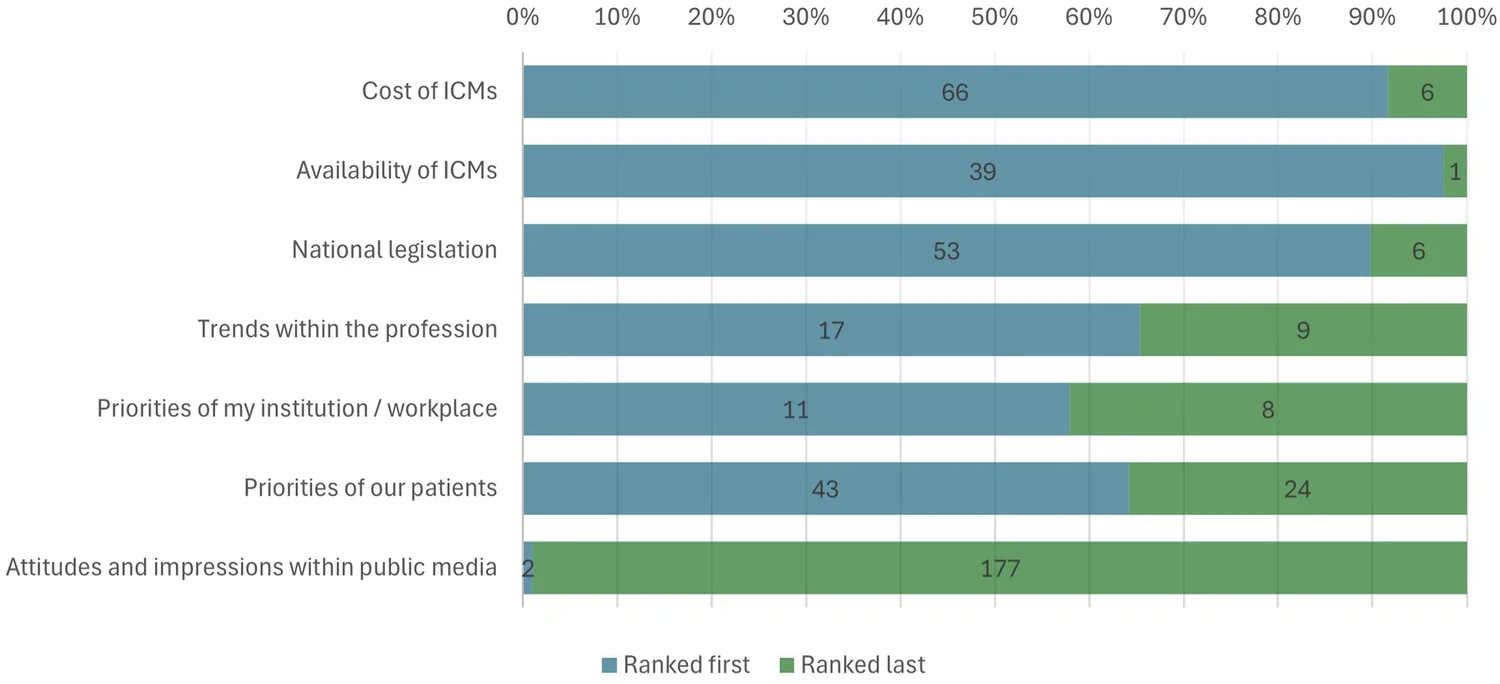
Factors ranked as most and least influential in shaping ICM sustainability practices

The primary drivers for sustainability included institutional support, regulatory compliance, and professional advocacy (Fig. 6). Respondents working in large public hospitals highlighted the crucial role of robust institutional frameworks, including well-defined policies, resource availability, and dedicated sustainability committees, in fostering sustainable practices. These institutions were noted for their ability to integrate sustainability into routine operations and strategic goals. Conversely, limited institutional support in smaller hospitals emerged as a significant barrier, often linked to budgetary constraints, insufficient training, and understaffing. Statistical analysis using a chi-square test revealed a significant association between institutional size and perceived drivers of sustainability $\left({X}^{2}\left(3,{n}=231\right)=30.34,{p} < 0.001\right)$, underscoring the disparities between large and small institutions. Regulatory compliance was frequently cited as a motivator, with professionals emphasising its influence on prioritising sustainability. Younger professionals were particularly vocal about professional advocacy, with an ANOVA test confirming significant differences in advocacy levels across age groups $\left(F\left(3,\,227\right)=4.89,{p}=0.003\right)$. Post hoc Tukey analysis showed that respondents aged 25–34 and 35–44 years reported significantly higher levels of advocacy compared with those aged 45–54 and ≥ 55 years $\left(p < 0.05\right)$, with no significant difference between the two younger age groups.

## Discussion

This international survey provides important insights into healthcare professionals’ KAPs concerning the sustainability of ICM. With responses from 231 participants across 44 countries, the study reveals encouraging awareness of sustainability issues but also highlights substantial variability in reduction strategies, waste management, and patient education. Notably, while 60% of respondents reported having institutional sustainability policies, one-quarter remained neutral, potentially suggesting either limited communication or weak policy enforcement. Furthermore, waste management practices were inconsistent, with 26% reporting no engagement at all, and nearly half of the respondents “strongly disagreed” that patients were aware of ICM’s environmental implications. Both individuals and professional bodies should consider how to increase awareness and engagement for those involved in ICM administration as well as for those who receive ICMs.

The growing demand for ICM has raised concerns regarding both resource availability and environmental impact. Global iodine reserves are finite, and growing demand for ICMs may place further challenges on supplies over the long-term [1]. Recent analyses have warned that rising consumption, coupled with dependence on finite raw materials, may lead to further shortages if sustainability measures are not prioritised [8]. At the same time, ICM residues have been widely detected in waste, surface and drinking waters, raising ecological and public health concerns. Dekker et al highlighted that ICM contamination is persistent and contributes to the formation of toxic iodinated disinfection by-products in drinking water systems [7]. Our survey findings align with these concerns, demonstrating that many professionals recognise the urgency of sustainable practices in ICM use. Awareness is only one part of a much-needed strategy to encourage effective ICM waste management.

Recent studies from Norway and China have shown that single-dose packaging and poor disposal practices contribute significantly to poor ICM waste management packages. Rusandu and colleagues reported that hospitals in Norway lacked consistent systems for ICM waste collection, echoing the variability highlighted in our survey [19]. Most, if not all, ICM manufacturers highlight the presence of iodine leftover recycling schemes; however, globally, these are not universally available, and uptake can be variable. Lindsey et al demonstrated that transitioning from single-use to multidose systems could reduce both financial and environmental costs of ICMs [12]. We found that large public hospitals report higher engagement in sustainable practices compared with smaller institutions, further supporting the observation that resource availability may play a pivotal role in implementation. Alternative explanations should also be considered; large hospitals would have greater opportunities for ICM recycling but would also receive greater economic benefits for participating in such a scheme.

Reduction strategies such as personalised protocols, saline chasers, and automated injectors were variably employed in our cohort. These approaches have been shown to reduce ICM usage without compromising diagnostic quality. For example, Wei et al demonstrated substantial reductions in costs and waste when using multidose packaging in CT departments [13]. However, the survey highlights limited uptake in smaller institutions, possibly suggesting the need for resource-sharing or incentive-based schemes to bridge this gap.

Perceptions regarding potential patient awareness emerged as particularly low. Nearly half of the respondents “strongly disagreed” that patients understood the environmental consequences of ICM use. This finding is consistent with the study by Zanardo et al, which found that patients displayed limited awareness of contrast-related environmental issues but expressed a strong interest in sustainable healthcare practices when informed [1]. This gap highlights an opportunity for professional bodies, such as the ESR and the EFRS, along with healthcare systems, to implement public-facing education campaigns that integrate sustainability into informed consent processes [20, 21].

Our results also demonstrate generational differences, with younger professionals (25–44 years) more engaged in ICM reduction and waste management initiatives. This aligns with broader evidence indicating that younger healthcare providers are more attuned to environmental issues and more likely to advocate for sustainable practices [22]. Such findings could suggest that educational initiatives targeting established professionals may be particularly valuable.

The findings underscore the need for stronger institutional frameworks and international collaboration. Large hospitals demonstrated higher adoption of sustainable practices, reflecting the benefits of scale and resources. Smaller hospitals and standalone facilities, however, require targeted support, including access to shared waste-management systems and sustainability training [23]. International radiology societies, such as the ESR and EFRS, are well-positioned to spearhead campaigns promoting best-practice guidelines and the harmonisation of sustainability strategies. An example of this is the opportunity from the ESR to submit an application for an ESR Green ID Certificate [24]. The aim is to recognise and support the commitment of imaging departments in implementing sustainable practices and play a central role in achieving the ESR’s sustainability goal [25].

Furthermore, embedding sustainability into procurement policies, accreditation frameworks and training curricula may provide a mechanism to standardise practices across institutions [26]. Similarly, engaging patients through education initiatives could improve acceptance of modified contrast protocols and support sustainable imaging pathways [16]. A potential for this comes in the patient information material typically provided when advising patients on examinations in CT, MRI and fluoroscopy.

Our study does, however, have several limitations. First, while the survey achieved a wide geographical reach, responses were disproportionately concentrated in a few countries, potentially limiting generalisability. Second, as with all surveys, self-reporting bias may have influenced responses, particularly concerning sustainability practices. Readers should interpret study findings with some caution, as those who responded to the survey may have had a prior interest in ICM sustainability. Finally, the sample size, though adequate for descriptive and comparative analyses, is considered insufficient to draw global generalisations, particularly in view of selection bias. It is not possible to know how many potential participants received the survey links and thus what the study response rate was. It should be noted that the time of the survey, membership of the ESR was reported to be around 140,000 and that the EFRS was representing over 100,000 radiographers. Future research should expand recruitment to underrepresented regions, incorporate longitudinal designs to assess evolving practices, and integrate objective data (e.g., institutional audits of ICM use and waste) to complement self-reported measures. It would also be of value to consider similar research investigating sustainability practices relating to the use of gadolinium-based contrast agents. Concerns regarding the availability of gadolinium have not, as yet, generated the same concerns as those for ICMs; however, the safety profile of gadolinium and the subsequent effects of contamination of waste and drinking water are under increasing scrutiny [7].

In terms of specific recommendations from this study,

1. International professional bodies, such as the ESR/EFRS, should consider opportunities to include contrast media sustainability within wider policy documents and when guiding training curricula.
2. Patient information literature should also be amended to highlight an institution’s mechanisms to ensure ICM sustainability.
3. Institutions should consider obtaining the ESR Green ID Certificate.
4. The ESR/EFRS, amongst others, should look at developing resources which would support the wider utilisation of ICM sustainability practices.

In conclusion, this survey demonstrates growing professional recognition of the importance of ICM sustainability but also exposes critical gaps in waste management, reduction strategies, and patient education. Variability across institutions, professions, and countries highlights the need for harmonised policies and resource-sensitive implementation strategies. Strengthening institutional frameworks, fostering international collaboration, and engaging patients and professionals alike are essential to safeguard ICM supply, mitigate environmental risks, and promote sustainable radiology practice.

## Supplementary information

Supplementary file

## Data Availability

A fully anonymised dataset of study data may be available to third parties for subsequent analysis. The authors will consider requests on an individual, case-by-case basis.

## Abbreviations

ANOVA: Analysis of variance
EFRS: European Federation of Radiographer Societies
ESR: European Society of Radiology
ICM: Iodinated contrast media
KAPs: Knowledge, attitudes, and practices
SUSTAINED: Sustainable iodine contrast media for a healthy future
