# Supplementary material for: Sustainable iodine contrast media for a healthy future (SUSTAINED): a global survey of practice
Source: Insights Imaging. 2026 Oct 11;17:249. doi: 10.1186/s13244-026-02392-4 (PMC13654907; doi:10.1186/s13244-026-02392-4)

Sustainable Iodine Contrast Media for a Healthy Future (SUSTAINED): A Global Survey of Practice

ELECTRONIC SUPPLEMENTARY MATERIAL

S1 - Survey Instrument

Sustainable Iodine Contrast Media Usage for a Healthy Future (SUSTAINED)

Start of Block: Demographics

Q1-How do you describe yourself?

- ○ Male (1)
- ○ Female (2)
- ○ Non-binary / third gender (3)
- ○ Prefer to self-describe (4) ____
- ○ Prefer not to say (5)

Q2-How old are you?

- ○ Under 18 (1)
- ○ 18-24 years old (2)
- ○ 25-34 years old (3)
- ○ 35-44 years old (4)
- ○ 45-54 years old (5)
- ○ 55-64 years old (6)
- ○ 65+ years old (7)

End of Block: Demographics

Start of Block: Country

Q3-In which country do you currently reside/practice/work?

▼ Afghanistan (1) ... Zimbabwe (1357)

Q4-From the following categories, what category best describes **your current role / profession**?

- ◯ Diagnostic Radiographer / Radiological Technologist (1)
- ◯ Therapeutic Radiographer / Radiation Therapist / Radiotherapist (2)
- ◯ Radiologist / Radiology Resident (3)
- ◯ Radiation Oncologist / Oncology Resident (4)
- ◯ Cardiologist / Cardiology Resident (5)
- ◯ Vascular Surgeon / Surgical Resident (6)
- ◯ Anaesthesiologist / Anaesthetist / Anaesthesia Resident (7)
- ◯ Other Medical Specialist (8)
- ◯ Other Allied Health Professional (9)
- ◯ Representative from Industry (10)
- ◯ None of the above (11)

Q5-Which of the following categories best describes **your workplace / institution**?

- ○ Large-size public hospital (>500 beds) (1)
- ○ Medium-size public hospital (100-500 beds) (2)
- ○ Small-size public hospital (3)
- ○ Large-size private hospital (>500 beds) (4)
- ○ Medium-size private hospital (100-500 beds) (5)
- ○ Small-size public hospital (6)
- ○ Combined public / private hospital (7)
- ○ Standalone imaging facility i.e. Community Diagnostic Hub (8)
- ○ Academic Centre i.e. University (9)
- ○ Other (please state) (10) ____

Q6-Which of the following area/areas of imaging / clinical practice do you **commonly work**? (*select all that apply*)

- ☐ General radiography (X-ray) (1)
- ☐ Operating Theatre / Department (2)
- ☐ Radiology-based fluoroscopy (non-intervention) (3)
- ☐ Interventional Radiology (4)
- ☐ Cardiology (Cath-Labs) (5)
- ☐ Computed tomography (CT) (6)
- ☐ Magnetic Resonance Imaging (MRI) (7)
- ☐ Nuclear medicine, including PET-CT (8)
- ☐ Radiation Therapy (9)
- ☐ Other (10) ____

End of Block: Country

Start of Block: Institutional Attitudes to ICM Sustainability

Q7-With respect to **your Institution / Workplace**, please rate the following statements.

	Strongly Agree (1)	Agree (2)	Neither Agree nor Disagree (3)	Disagree (4)	Strongly Disagree (5)	Not applicable / don't know (6)
Iodinated contrast media sustainability is a priority within my workplace. (1)	○	○	○	○	○	○
We have a contrast media conservation strategy at an INSTITUTIONAL level. (2)	○	○	○	○	○	○
We have a contrast media conservation strategy at an DEPARTMENTAL level. (3)	○	○	○	○	○	○
We have a contrast media conservation strategy at an MODALITY level. (4)	○	○	○	○	○	○
Giving the lowest possible iodine dose to a patient is a priority. (5)	○	○	○	○	○	○
Recycling waste iodinated contrast is a priority. (6)	○	○	○	○	○	○
Safe and responsible disposal of iodinated contrast is a priority. (7)	○	○	○	○	○	○
Iodinated contrast shortages will become more of a problem in future years. (8)	○	○	○	○	○	○

Safe and responsible disposal of waste iodinated contrast media will become more important in future years. (9)	○	○	○	○	○	○
Alternatives to iodinated contrast media are urgently needed in healthcare. (10)	○	○	○	○	○	○

End of Block: Institutional Attitudes to ICM Sustainability

Start of Block: Institutional strategies to reduce iodinated contrast usage.

Q8-With respect to your practice / institution / workplace, which of the following iodinated contrast media **reduction strategies** do you currently utilise?

	Always (1)	Most of the time (2)	About half the time (3)	Sometimes (4)	Never (5)
Personalised individualised patient' contrast protocols. (1)	○	○	○	○	○
Use of saline chaser bolus. (2)	○	○	○	○	○
Consideration of alternative imaging modalities where possible i.e. MRI. (3)	○	○	○	○	○
Optimisation of imaging parameters i.e. low kV selection in CT. (4)	○	○	○	○	○
Re-use of ICM leftovers. (5)	○	○	○	○	○
Use of multi-patient contrast delivery systems. (6)	○	○	○	○	○

End of Block: Institutional strategies to reduce iodinated contrast usage.

Start of Block: Contrast and Consumable Waste Management

Q9-Which of the following practices would be undertaken in your institution / workplace?

	Always (1)	Most of the time (2)	About half the time (3)	Sometimes (4)	Never (5)
Correct disposal of contrast leftovers. (1)	○	○	○	○	○
Collection and handling of contrast excretions. (2)	○	○	○	○	○
Environmental handling of waste consumables and packaging. (3)	○	○	○	○	○
Use of multi-patient ICM administration systems. (4)	○	○	○	○	○
Periodic reporting of contrast media (ICM) consumption. (5)	○	○	○	○	○
Periodic reporting of contrast media (ICM) waste management results. (6)	○	○	○	○	○
Periodic reporting of ICM related recycling results. (7)	○	○	○	○	○

End of Block: Contrast and Consumable Waste Management

Start of Block: Patient Awareness

Q10-With respect to **patients** within your workplace / institution, how would you rate the following statements?

	Strongly Agree (1)	Agree (2)	Neither Agree / Disagree (3)	Disagree (4)	Strongly Disagree (5)
Patients are aware of institutional processes to minimise ICM consumption. (1)	○	○	○	○	○
Patients are aware of institutional processes to correctly manage ICM leftovers and excretions. (2)	○	○	○	○	○
Sustainable ICM practices are important to our patients. (3)	○	○	○	○	○
Patients believe that sustainable and environmentally responsible healthcare is very important. (4)	○	○	○	○	○

End of Block: Patient Awareness

Start of Block: Priorities

Q11-Please rank the following factors in ***order of importance*** regarding *drivers* for improving ICM sustainability.

_______ Cost of ICMs (1)
_______ Availability of ICMs (2)
_______ National Legislation (3)
_______ Trends within the Profession (4)
_______ Priorities of My Institution / Workplace (5)
_______ Priorities of Our Patients (6)
_______ Attitudes and Impressions within Public Media (7)

End of Block: Priorities

Figure S2. Graphical illustration of the characteristics of survey respondents (n=231).

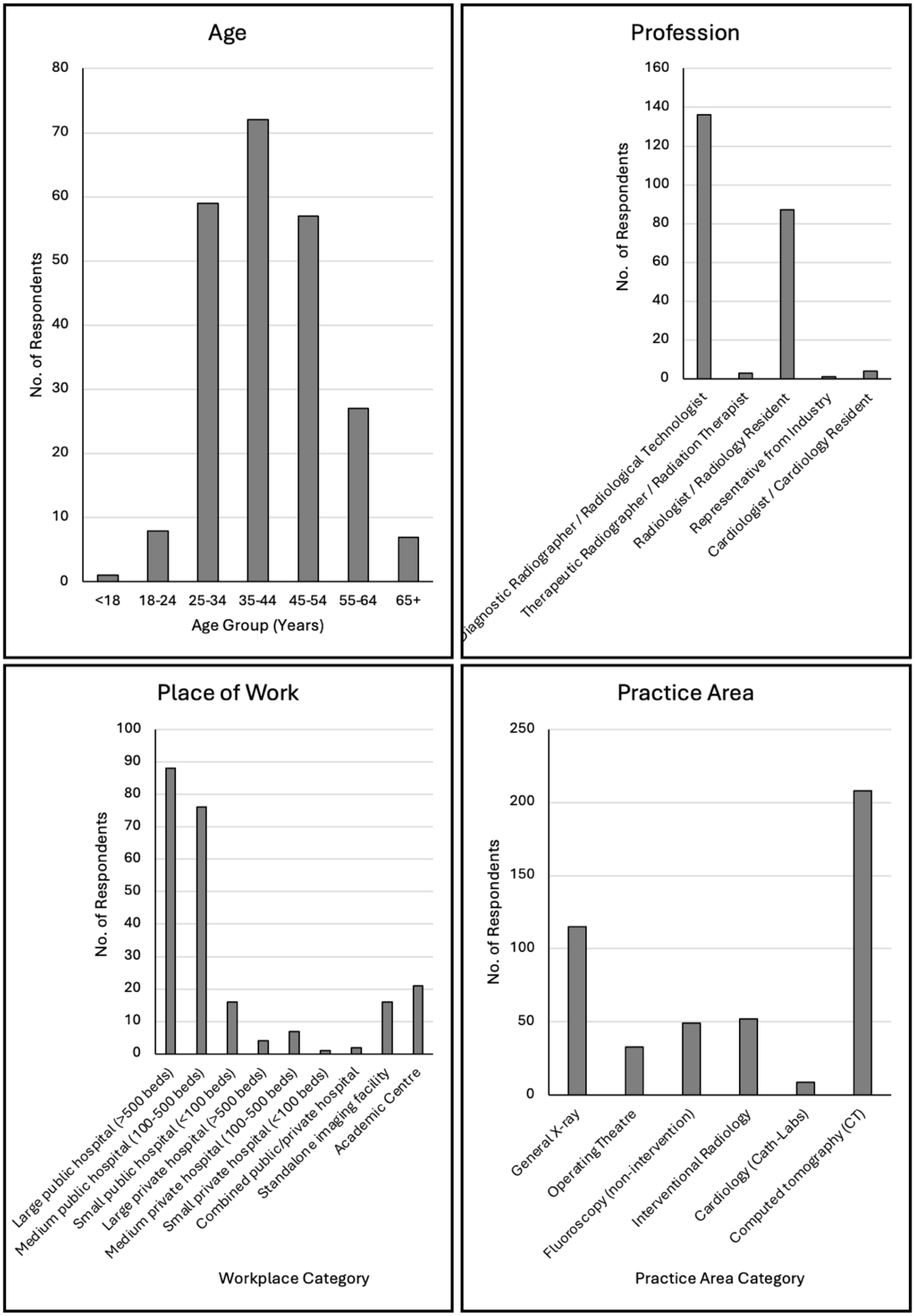

Supplement: Supplementary file 1 [file 13244_2026_2392_MOESM1_ESM.pdf]
